# A new biomarker to predict atrial fibrillation and its adverse events after coronary artery bypass surgery: red blood cell distribution volume

**DOI:** 10.5830/CVJA-2022-063

**Published:** 2023-01-11

**Authors:** Hüseyin Şaşkın, Durmuş Alper Görür

**Affiliations:** Health Sciences University, Derince Training and Research Hospital, Kocaeli, Turkey

**Keywords:** coronary artery bypass grafting surgery, red blood cell distribution volume, atrial fibrillation

## Abstract

**Objective:**

Recent studies suggest that increased red blood cell distribution width may be associated with increased risk of atrial fibrillation. This study aimed to evaluate the relationship between pre-operative and postoperative erythrocyte distribution volume, postoperative atrial fibrillation and related adverse events in patients undergoing isolated coronary artery bypass surgery.

**Methods:**

A total of 790 patients (611 males, mean age 58.3 ± 6.2 years) in pre-operative sinus rhythm, who underwent isolated coronary artery bypass grafting with cardiopulmonary bypass at the same centre and by the same surgical team between January 2015 and December 2021, were enrolled retrospectively. Two groups were created, group 1 (n = 183) and group 2 (n = 607), with regard to the occurrence of atrial fibrillation in the early postoperative period or not, respectively. Clinical and demographic data, biochemical and complete blood count parameters, and intra-operative and postoperative data of the patients were recorded. Univariate and subsequent multivariate logistic regression analysis was done to determine significant clinical factors and independent predictors of postoperative atrial fibrillation.

**Results:**

Among the patients, 182 (23.2%) developed atrial fibrillation during the 72 hours postoperatively. Pre-operative and postoperative first-, third- and seventh-day red blood cell distribution volume (p = 0.0001), C-reactive protein (p = 0.0001) and erythrocyte sedimentation rate (p = 0.0001) were significantly increased in group 1. Multivariate logistic regression analysis showed elevated pre-operative and postoperative first-, thirdand seventh-day red blood cell distribution volume, erythrocyte sedimentation rate and C-reactive protein as independent predictors of early postoperative atrial fibrillation.

**Conclusion:**

Pre-operative and postoperative red blood cell distribution volume was found to be an independent predictor of atrial fibrillation and associated adverse events in the early postoperative period of isolated coronary artery bypass grafting.

Coronary artery bypass grafting (CABG) is an effective coronary revascularisation method increasingly used in the treatment of coronary artery disease (CAD).^1^ As a result of developments in cardiopulmonary bypass (CPB) technology, CABG can be performed with lower mortality and morbidity rates.^2^

Postoperative atrial fibrillation (POAF), the most common arrhythmic sequel of cardiac surgery, is a serious complication that has plagued postoperative management for decades.^3^ The incidence of POAF varies according to the type of surgery and is seen in 3% of unselected adults aged 45 years and older who undergo non-cardiac surgery, 30% after thoracic surgery, and 20–50% especially after valve and bypass surgery.^4^ Mahoney et al., in their retrospective study involving 10 550 patients, reported that POAF developed in 33.8% of patients after CABG and heart valve surgery.^5^

Studies have shown that POAF is associated with many factors, including valvular heart disease, gender, age, obesity, obstructive sleep apnoea, chronic obstructive pulmonary disease and increased epicardial adipose tissue.^6^ Age is the only systematically proven risk factor for POAF in the literature, and findings regarding other possible pre-operative risk factors are being discussed.^5^

The exact pathophysiology of POAF is unclear, and there are numerous studies showing that its onset and development are associated with inflammation, oxidative stress, cardiac ischaemia and sympathetic activation.^7^ POAF not only increases the length of hospital stay and hospitalisation costs, but also increases the incidence of peri-operative complications, stroke and mortality, leading to a poor prognosis, but its long-term effects are not fully known.^8^ Therefore, it is very important to find specific biomarkers to predict, prevent and treat POAF before poor outcomes after cardiac surgery.^9^

Because biomarkers are dynamic, their inclusion in the management of patients with AF is highly recommended.^10^ Unfortunately, an accurate scoring tool has not been defined for estimating POAF.^5^ The search for haematological determinants of AF began in 1987, reported in a publication by Imataka et al., which showed significant impairment of plasma volume and erythrocyte biology in patients with AF.^11^

Various inflammatory biomarkers including white blood cell (WBC) count, leukocyte subtypes, platelets, mean platelet volume (MPV), C-reactive protein (CRP), neutrophil-tolymphocyte ratio (NLR) and platelet-to-lymphocyte ratio (PLR) have been reported to be important prognostic determinants in cardiovascular disease.^12^ Previous studies have reported the association of mediators such as CRP, tumour necrosis factor-α (TNF-α), interleukin 2 (IL-2), IL-6, IL-8 and monocyte chemoattractant protein 1 (MCP-1) with POAF.^13^ Recently, AF has been associated with routine biomarkers of inflammation and oxidative stress, such as uric acid, red blood cell distribution width (RDW) and γ‐glutamyl transferase (γ-GT).^14^

Given the problem associated with advanced age, combined co-morbid status and subsequent risk stratification of patients presenting for CABG, RDW is increasingly being sought for the prediction of postoperative outcomes.^15^ RDW from a standard complete blood count is a convenient and inexpensive biochemical parameter that represents the size variability of circulating erythrocytes.^14^ Increased RDW indicates the presence of anisocytosis, which reflects chronic inflammation and high levels of oxidative stress, is associated with impaired erythropoiesis and erythrocyte destruction.^15^

Along with its extensive use in diagnostic haematology, RDW has been associated with the presence and complications of a wide variety of human pathologies, including cardiovascular disease, over the past decade.^13^ Although the link between RDW and cardiovascular diseases has not been fully defined, previous studies have shown that RDW correlates with markers of chronic inflammation (such as IL-6, IL-8, IL-12, IL-17, IL-18, interferon and TNF).^15^

Kılıcgedik et al. reported in their retrospective study that increased RDW values were a predictor of AF developing after CABG.^16^ Likewise, Gecmen et al. found in their study with 94 patients operated on for isolated CABG surgery with CPB, who were followed up until discharge from the cardiovascular intensive care unit (ICU), that RDW independently predicted the risk of POAF.^17^

Previous studies have attempted to demonstrate the effect of inexpensive, simple and routinely measured RDW on predicting the onset of POAF. However, the prognostic value of RDW after AF in critically ill patients who underwent isolated CABG with CPB is largely unknown. The aim of our study was to predict the development of POAF in critically ill patients and investigate the potential clinical importance of RDW in predicting in-hospital mortality and morbidity in those with AF.

## Methods

The medical records of a total of 1 096 patients who underwent isolated CABG between January 2015 and December 2021 were reviewed retrospectively. The operations were performed at the same centre by the same surgical team.

We enrolled 790 (72.1%) patients with pre-operative normal sinus rhythm who underwent isolated CABG with CPB. Two different populations were created: group 1 (n = 183) including patients who developed AF in the first 72 hours postoperatively; and group 2 (n = 607), including patients who remained in normal sinus rhythm. AF diagnosis was based on the criteria proposed by the American Heart Association/American College of Cardiology/Heart Rhythm Society (AHA/ACC/HRS) 2019 guidelines for AF.^18^ AF was defined as the demonstration of AF for a minimum duration of 30 seconds using electrocardiography (ECG) recordings.

Patients with pre-operative non-sinus rhythm, a history of paroxysmal or chronic AF, an implanted cardiac device or electrophysiological ablation were excluded from the study. In addition, also excluded from the study were patients who had valvular heart disease, systemic inflammatory diseases, chronic obstructive pulmonary disease, malignancy, haematological proliferative diseases, autoimmune diseases, endocrinological disorders, left ventricular systolic function disorder (left ventricular ejection fraction ≤ 30%), advanced age (> 75 years), chronic kidney disease, patients with low pre-operative haemoglobin levels (≤ 10 g/dl), patients with a left atrial diameter > 4.5 cm in echocardiography, the presence of signs of clinical infection [fever > 37.5°C, CRP ≥ 5 mg/dl, erythrocyte sedimentation rate (ESR) > 20 mm/h or leukocyte count > 11 000 cells/μl] before surgery, emergency operations, patients who required intra-aortic balloon pump, patients who were re-operated on due to haemodynamic instability or bleeding, patients who were operated on a beating heart or redo CABG.

Demographic data, pre-operative–postoperative clinical and biochemical parameters, and intra-operative and operative information of the patients were obtained retrospectively from the hospital’s software system. We also recorded age, gender, smoking (defined as continuous or cumulative smoking for ≥ six months or at least six months every day; passive smoking, which refers to non-smokers inhaling the smoke from smokers’ breath for at least 15 minutes per day for more than one day per week), history of statin use, diabetes (including history of diabetes mellitus or newly diagnosed diabetes), hypertension (defined as history of hypertension or newly diagnosed hypertension), dyslipidaemia [defined as low high-density lipoprotein cholesterol (HDL-C) and high triglyceride levels; the cut-off values were selected at HDL-C < 40 mg/dl (1.04 mmol/l) and triglycerides ≥ 200 mg/dl (2.26 mmol/l) in both men and women), left ventricular ejection fraction (LVEF), left atrial diameter, laboratory parameters (haemoglobin, haematocrit, leukocyte count, thrombocyte count, RDW, and total cholesterol, triglyceride, serum creatinine, urea, ESR and CRP levels), operation information, the number of grafts used, duration of CPB and aortic cross-clamp (ACC), use of blood products and length of stay in the ICU and hospital.

The pre-operative basal heart rates of the patients were obtained by analysing 12-lead ECG records. At days one, three and seven after surgery, additional ECGs and blood samples were obtained for standardised postoperative follow up.

Fasting venous blood samples were routinely obtained from patients, both pre-operatively and on the first, third and seventh postoperative days for complete blood count (CBC), CRP and ESR analysis. Approximately 5 to 7 ml venous blood samples were placed into a sterile tube with EDTA. Haematological parameters were calculated by an automated blood count device (Abbott CELL-DYN 3700; Abbott Laboratory, Abbott Park, Illinois, USA) following a waiting time of one hour.

Serum levels of total cholesterol, low-density lipoprotein cholesterol (LDL-C), HDL-C and triglycerides were determined in all specimens using an automatic multichannel biochemical analyser (Hitachi-7450, Hitachi, Tokyo, Japan) following routine laboratory procedures. Levels of CRP were determined using immune-enhanced nephelometry.

The patients were followed in the ICU and in-patient room with continuous ECG monitoring for the first 48 hours. In the absence of any contra-indication, oral 50 mg/day metoprolol was started in all patients on the first postoperative day. During the in-patient clinic follow up of the patient’s rhythm ECG, pulse and arterial blood pressure measurements were performed at maximum intervals of four hours. A patient’s complaint of cardiac arrhythmia or palpitation was detected by standard 12-lead ECG recording. A diagnosis of AF was made with ECG recording.

Intravenous (IV) metoprolol (5–10 mg) was administered to control heart rate in the treatment of AF. All required patients were given a 300-mg IV bolus of amiodarone within one hour and continuous 900-mg IV amiodarone over the next 24 hours for maintenance, followed by 200 mg of amiodarone orally three times a day. During the entire AF period, low-molecular-weight heparin was administered (enoxaparin 0.1 mg/kg twice daily). Patients who did not return to sinus rhythm were discharged on oral warfarin therapy.

This study complied with the Declaration of Helsinki and was carried out following approval of the Ethics Committee for Clinical Trials of Kocaeli Derince Training and Research Hospital of Health Sciences University (approval number: 2022- 105).

All patients were operated on with median sternotomy under general anaesthesia. Standard CPB was established with aortic and venous cannulations, and systemic heparin (300 IU/kg) was administered, with the maintenance of activated clotting time > 450 seconds. Hyperkalaemic cold blood cardioplegia was applied for cardiac arrest. Surgery was performed under moderate hypothermia (28–30oC). CPB flow was maintained at 2.2–2.5 l/min/m^2^, mean perfusion pressure between 50 and 80 mmHg, and haematocrit level between 20 and 25%.

After distal anastomoses were made, the aortic cross-clamp was removed and proximal anastomoses were done on the ascending aorta under a lateral clamp on a beating heart. CPB was terminated following stable haemodynamics. Following decannulation, the heparin was neutralised with protamine.

All patients were transferred postoperatively to the ICU intubated. They were extubated following the onset of spontaneous breathing and normalisation of orientation and co-operation if the haemodynamic and respiratory functions were appropriate.

## Statistical analysis

IBM SPSS statistics version 22.0 software (SPSS Inc, Chicago, IL) was used for the analysis of data. Descriptive data are presented as number (percentage), mean ± standard deviation, or median (range), where appropriate. Categorical variables were compared using Pearson’s chi-squared test or Fisher’s exact test. Depending on the normality of the data, continuous variables were compared using the Mann–Whitney U- or student’s t-test for independent samples. Among the data measured, the normality of distribution was evaluated by histogram or Kolmogorov–Smirnov test, and the homogeneity of distribution was evaluated by ‘Levene’s test for equality of variance’.

The effects of the risk factors suggested to be influential on the early postoperative AF were studies with univariate logistic regression analysis. The multiple effects of the risk factors that were influential, or were suggested to be influential in predicting the early postoperative AF as a result of the univariate statistical analysis were studied through the retrospective selective multivariate logistic regression analysis. The odds ratio, 95% confidence interval (CI) and significance level for each of the risk factors were found statistically significant for p < 0.05.

## Results

The demographic characteristics and clinical data of the patients are summarised in Table 1. There were no differences between the two groups in terms of demographic and clinical data.

**Table 1:** Demographic and clinical properties of the patients

*Patients' characteristics*	*Group 1 with AF (n = 183)*	*Group 2 without AF (n=607) =*	p-value
Age, years (mean + SD)	58.2 + 5.9	58.3 + 6.3	0.81**
Male, n (%)	(77.6)	469 (77.3)	0.93*
Female, n (%)	(22.4)	138 (22.7)	
Hypertension, n (%)	54 (19.9)	129 9 (24.9)	0.12*
Diabetes mellitus, n (%)	2 (20.2)	131 (24.6)	0.18*
Smoking, n (%)	5 (24.3)	08 (22.5)	0.56*
Hyperlipidaemia, n (%)	83 (22.9)	100 (23.1)	0.89*
BMI (kg/m²) (mean + SD)	26.5 + 3.7	26.3 + 3.5	0.67**
Ejection fraction (%) (mean + SD)	54.8 + 8.8	55.1 + 8.9	0.68**
Basal heart rate (bpm) (mean + SD)	65.8 + 7.4	66.6 + 7.3	0.18**
Left atrial diameter (mm) (mean + SD)	34.8 + 3.4	34.5 + 3.1	0.25**

AF: atrial fibrillation, BMI: body mass index.

*Pearson’s chi-squared test,**Student’s *t*-test.

The pre-operative blood analysis and haematological parameters of the patients are summarised in Table 2. RDW (p = 0.0001), ESR (p= 0.0001) and CRP (p= 0.0001) levels were significantly different between the groups.

**Table 2:** Pre-operative blood results and haematological parameters of patients

*Pre-operative blood results and haematological parameters*	*Group 1 with AF (n=183)*	*Group 2 with AF (n=607)*
	*Mean + SD*	*Mean + SD*	p-value
Haemoglobin (mg/dl)	13.6 + 1.4	13.3 + 1.5	0.11*
Creatinine (mg/dl)	0.73 + 0.30	0.75 + 0.29	0.92*
Urea (mg/dl)	40.7 + 3.9	40.6 + 3.8	0.90*
HbA, (%)	6.2 + 1.5	6.3 + 1.4	0.58*
Leucocyte counts (x 10 ³ cells/ul)	8.1 + 1.5	8.2 + 1.6	0.81*
Thrombocyte counts (x 10 cells/ul)	253 + 60	261 + 62	0.10*
CRP (mg/l)	1.53 + 0.63	0.63 + 0.38	0.0001*
ESR (mm/h)	12.13 + 3.41	7.57 + 3.21	0.0001*
RDW (%)	15.40 + 0.92	13.69 + 1.09	0.0001*
HDL-C (mg/dl) (mmol/l)	35.9 + 5.9 0.93 + 0.15	36.7 + 6.5 0.95 + 0.17	0.17*
LDL-C(mg/dl) (mmol/l)	124.9 + 18.3 3.23 + 0.47	126.9 + 17.5 2.29 + 0.45	0.21*
Total cholesterol (mg/dl) (mmol/l)	189.2 + 40.7 4.9 + 1.05	187.2 + 40.3 4.73 + 1.04	0.56*
Triglycerides (mg/dl)	155.3 + 67.5	159.4 + 76.7	0.51*
(mmol/l)	1.75 + 0.76	1.8 + 0.87	

AF: atrial fibrillation, HDL-C: high-density lipoprotein cholesterol, LDL-C: low-density lipoprotein cholesterol, HbA1c: glycated haemoglobin, CRP: C-reactive protein, ESR: erythrocyte sedimentation rate, RDW: red blood cell distribution width.

*Student’s *t*-test.

The early postoperative blood analysis and haematological parameters of the patients are summarised in Table 3. Postoperative first-, third- and seventh-day CRP (p = 0.0001), ESR (p = 0.0001) and RDW (p = 0.0001) levels were significantly different between the groups.

**Table 3:** Early postoperative blood results and haematological parameters of patients

*Early postoperative blood results and haematological parameters*	*Group 1 with AF (n = 183) Mean + SD*	*Group 2 without AF (n 607) Mean + SD*	p-value
Haemoglobin (mg/dl)			
Day 1	9.02 + 1.03	9.19 + 1.13	0.07*
Day 3	10.13 + 1.73	10.07 + 1.59	0.51*
Day 7	10.79 + 1.87	10.83 + 1.91	0.48*
Leukocyte counts (X 103 cells/ul)			
Day 1	13.11 + 3.32	13.18 + 3.40	0.43*
Day 3	11.73+3.07	11.64+3.09	0.72*
Day 7	10.13 + 2.81	10.09 + 2.89	0.64*
CRP (mg/l)			
Day 1	36.59 + 9.03	28.01 + 5.41	0.0001*
Day 3	33.89 + 10.40	31.03 + 7.84	0.0001*
Day 7	30.46 + 5.71	27.51 + 4.57	0.0001*
ESR (mm/h)			
Day 1	37.94 + 16.08	30.89 + 14.55	0.0001*
Day 3	49.96 + 9.71	41.36 + 6.40	0.0001*
Day 7	31.00 + 8.21	27.28 + 6.18	0.0001*
RDW (%)			
Day 1	15.13 + 1.50	13.38 + 1.44	0.0001*
Day 3	14.90 + 1. 30	13.40 + 1.40	0.0001
Day 7	14.44 + 0.82	12.87 + 1.18	0.0001*

AF: atrial fibrillation, CRP: C-reactive protein, ESR: erythrocyte sedimentation rate, RDW: red blood cell distribution width.

*Student’s *t*-test.

Among 790 patients, AF occurred in 183 (23.2%) subjects in the first 72 postoperative hours, and 163 (89.1%) of these returned to normal sinus rhythm with amiodarone therapy. Fourteen patients (7.6%) were discharged following conversion to sinus rhythm with electrical cardioversion; six patients (3.3%), for whom appropriate rate control could not be established, were discharged with AF rhythm and oral anticoagulant (warfarin sodium) therapy.

AF occurred in the first eight postoperative hours in 45 patients (24.6%); between nine and 24 hours in 52 patients (28.4%); between 25 and 72 hours in 69 patients (37.7%) and after 72 hours in 17 patients (9.3%).

The intra-operative and postoperative data of the patients are shown in Table 4. The length of stay in the ICU (p = 0.0001) and in hospital (p = 0.0001) were significantly different between the groups.

**Table 4:** Early postoperative blood results and haematological parameters of patients

	*Group 1 with AF (n = 183)*	*Group 2 without AF (n=607)*	
*Characteristics*	*Mean + SD*	*Mean + SD*	p-value
Aortic cross-clamp time (min)	52.6 + 13.5	51.9 + 13.2	0.56*
Cardiopulmonary bypass time (min)	83.1 + 16.4	82.2 + 16.6	0.52*
Number of anastomoses	3.35 + 1.04	3.34 + 0.98	0.90*
Amount of drainage (ml)	349 + 134	351 + 128	0.88*
Intubation time (h)	5.74 + 1.51	5.59 + 1.46	0.24*
Stay in the intensive care unit (h)	29.38 + 8.87	21.18 + 2.37	0.0001*
Total duration of hospital stay (d)	8.91 + 2.39	5.94 + 1.44	0.0001*
Use of blood products, n (%)	69 (37.7)	202 (33.3)	0.27**
Use of inotropic support, n (%)	17 (9.3)	36 (5.9)	0.11**

AF: atrial fibrillation, HDL-C: high-density lipoprotein cholesterol, RDW: red blood cell distribution width, LDL-C: low-density lipoprotein cholesterol, CRP: C-reactive protein, ESR: erythrocyte sedimentation rate, HbA1c: glycated haemoglobin.

The neurological event rate (transient ischaemic event, speech disorder, hemiplegia or hemiparesia) within the postoperative first month was significantly higher in group 1 compared to group 2 [10 patients (5.5%) vs six patients (1.0%); p = 0.001].

 Mortality in the first postoperative month occurred in five patients (2.7%) in group 1 and in two patients (0.5%) in group 2, which showed a statistically highly significant difference between the groups (p = 0.008).

The results of univariate and multivariate regression analysis of patients who developed AF in the early postoperative period are shown in Tables 5 and 6. In multivariate analysis, the variables that were found to be statistically significantly associated with POAF in univariate analysis were increased pre-operative RDW (p = 0.0001), CRP (p = 0.0001) and ESR (p = 0.0001) levels; increased postoperative first-, third- and seventhday RDW (p = 0.0001) levels; postoperative first-, third- and seventh-day ESR (respectively; p = 0.03, p = 0.0001, p = 0.03), and CRP (respectively; p = 0.0001, p = 0.04, p = 0.0001) levels. These were found to be independent predictors of early POAF.

**Table 5:** Univariate and multivariate regression analysis of pre-operative risk factors for postoperative AF

		*Postoperative*		*AF*	
*Variables*	*Unadjusted OR (95% CI)*	*p-value*	*R2*	*Adjusted OR (95% CI)*	p-value
Haemoglobin	1.10 (0.98-1.23)	0.11	-	-	-
Creatinine	0.98 (0.55-1.72)	0.92	-	-	-
Urea	1.01 (0.96-1.05)	0.89	-	-	-
HbA 1c	0.96 (0.86-1.08)	0.54	-	-	-
Left atrial diam- eter	1.03 (0.98-1.08)	0.28	-		
Ejection fraction	0.99 (0.97-1.01)	0.68	-		
Leucocyte counts	0.97 (0.91-1.03)	0.81	-	-	-
Thrombocyte counts	0.94 (0.81-1.07)	0.10	-	-	-
CRP	28.99 (17.78-42.27)	0.0001	0.42	15.35 (6.08-38.74)	0.0001
ESR	1.51 (1.40-1.62)	0.0001	0.26	1.55 (1.31-1.84)	0.0001
HDL-C	0.98 (0.95-1.01)	0.17	-	-	-
LDL-C	0.95 (0.91-0.99)	0.21	-	-	-
Total cholesterol	1.01 (0.98-1.04)	0.56	-	-	-
Triglyceride	0.97 (0.86-1.08)	0.51	-	-	-
RDW	5.24 (4.07-6.76)	0.0001	0.32	4.14 (2.35-7.31)	0.0001

AF: atrial fibrillation, HDL-C: high-density lipoprotein cholesterol, RDW: red blood cell distribution width, LDL-C: low-density lipoprotein cholesterol, CRP: C-reactive protein, ESR: erythrocyte sedimentation rate, HbA1c: glycated haemoglobin.

**Table 6:** Univariate and multivariate regression analysis of peri-operative and postoperative risk factors for postoperative AF

	*Postoperative AF*				
*Variables*	*Unadjusted OR (95% CI)*	*p-value*	*R2*	*Adjusted OR (95%*	p-value
Haemoglobin					
Day 1	0.86 (0.74-1.01)	0.07	-	-	-
Day 3	1.02 (0.96-1.08)	0.22	-	-	-
Day 7	0.96 (0.91-1.01)	0.27		-	-
Leukocyte counts					
Day 1	0.98 (0.96-1.00)	0.18	-	-	-
Day 3	0.93 (0.89-0.97)	0.72	-	-	-
Day 7	0.99 (0.91-1.07)	0.82	-	-	-
CRP					
Day 1	1.19 (1.15-1.23)	0.0001	0.24	1.31 (1.19-1.45)	0.0001
Day 3	1.04 (1.02-1.06)	0.0001	0.19	1.03 (0.95-1.12)	0.04
Day 7	1.12 (1.09-1.16)	0.0001	0.16	1.29 (1.14-1.44)	0.0001
ESR					
Day 1	1.03 (1.02-1.04)	0.0001	0.14	1.08 (1.01-1.15)	0.03
Day 3	1.14 (1.12-1.16)	0.0001	0.20	1.04 (1.01-1.07)	0.0001
Day 7	1.08 (1.05-1.11)	0.0001	0.15	1.09 (1.01-1.17)	0.03
RDW					
Day 1	2.54 (2.15-2.99)	0.0001	0.21	2.54 (1.63-3.90)	0.0001
Day 3	2.60 (2.19-3.10)	0.0001	0.19	2.65 (1.49-4.70)	0.0001
Day 7	4.61 (3.59-5.93)	0.0001	0.28	3.17 (1.74-5.77)	0.0001
Aortic cross-clamp time	1.01 (0.99-1.02)	0.56	-	-	-
Number of anasto- moses	1.01 (0.86-1.19)	0.90	-	-	-
CPB time	1.04 (0.92-1.16)	0.52	-	-	-
Intubation time	1.06 (0.96-1.16)	0.34	-	-	-
Use of blood prod- ucts	0.85 (0.59-1.17)	0.27	-	-	-
Use of inotropic support	0.62 (0.34-1.25)	0.12	-	-	-
Amount of drainage	0.97 (0.92-1.02)	0.89	-	-	-

CPB: cardiopulmonary bypass, CRP: C-reactive protein, ESR: erythrocyte sedimentation rate, AF: atrial fibrillation, RDW: red blood cell distribution width.

**Fig. 1:**
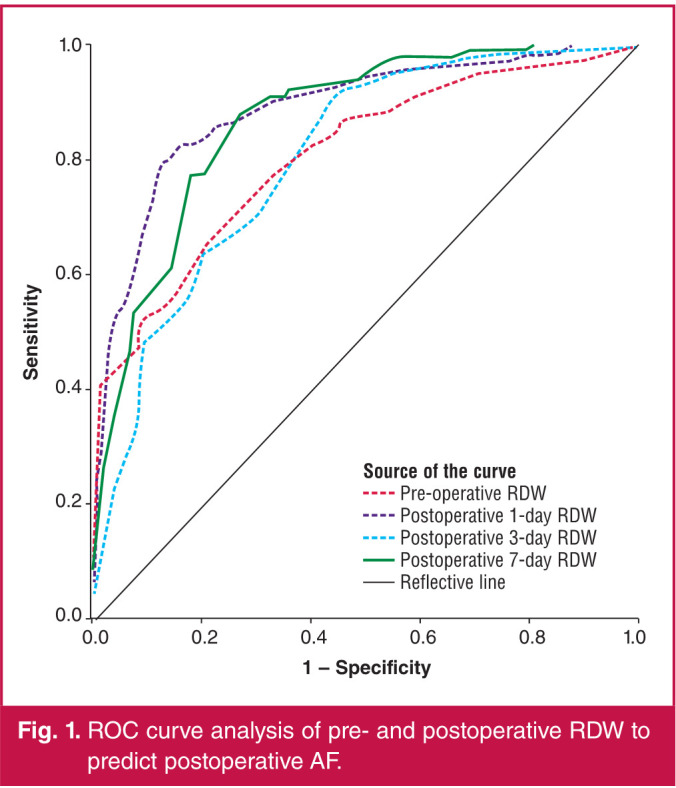
ROC curve analysis of pre- and postoperative RDW to predict postoperative AF.

The receiver operating characteristic (ROC) curves for pre-operative and postoperative first-, third- and seventh-day RDW were in association with POAF following isolated CABG (Fig. 1). The area under the curve (AUC) for the pre-operative RDW was 0.885 (95% CI: 0.856–0.915; p = 0.0001). Using a cut-off value of 14.55, the pre-operative RDW predicted POAF with a sensitivity of 83.1% and specificity of 80.2%.

The AUC for the postoperative first-day RDW was 0.805 (95% CI: 0.767–0.843; p = 0.0001). Using a cut-off value of 14.35, the postoperative first-day RDW predicted POAF with a sensitivity of 77.2% and specificity of 72.7%. The AUC for the postoperative third-day RDW was 0.802 (95% CI: 0.769–0.835; p = 0.0001). Using a cut-off value of 14.15, the postoperative third-day RDW predicted POAF with a sensitivity of 77.1% and specificity of 69.9%. The AUC for the postoperative seventh-day RDW was 0.865 (95% CI: 0.837–0.893; p = 0.0001). Using a cut-off value of 13.85, the postoperative seventh-day RDW predicted POAF with a sensitivity of 83.1% and specificity of 75.9%.

The pre-operative RDW level of the patients who underwent isolated CABG and died within one month postoperatively was 15.35 ± 0.94%, while it was 13.74 ± 0.78% for the patients who survived, which was statistically significantly different (p = 0.0001). Likewise, postoperative first-, third- and seventh-day RDW levels were significantly different between patients who died (respectively; 15.84 ± 0.95, 14.93 ± 0.87, 14.58 ± 0.82%) and who survived (respectively; 14.58 ± 0.86, 13.87 ± 0.79, 13.68 ± 0.71%) (p = 0.0001).

Moreover, the pre-operative RDW level was 15.54 ± 0.97% in patients who developed neurological events within one month postoperatively, and 13.62 ± 0.75% in patients who did not, which showed a statistically significant difference (p = 0.0001). Also, the RDW levels of the patients who developed neurological events on the first, third and seventh days postoperatively (respectively; 15.72 ± 0.91, 14.76 ± 0.84, 14.42 ± 0.78%) compared to those who did not (respectively; 14.66 ± 0.81, 13.72 ± 0.77, 13.54 ± 0.70%) were statistically significantly different (p = 0.0001).

## Discussion

In this study, we found that increased RDW in the pre-operative and early postoperative period was associated with early POAF and adverse events after isolated CABG. Again, we found a relationship between the increase in CRP and ESR levels, which are other easily measurable routine markers of inflammation in the pre-operative and early postoperative period, and early POAF after isolated CABG. In addition, we found that increased RDW and increased CRP and ESR levels in the pre-operative and early postoperative period were also valuable in predicting the development of POAF. To our knowledge, this is not the first study to evaluate the relationship between RDW and AF after CABG surgery but one of the few studies to demonstrate the relationship of RDW with the adverse effects of POAF.

Despite advanced treatment options, CABG remains the gold-standard treatment method for the treatment of patients with multivessel coronary artery disease.^2^ CABG is a high-risk procedure and many patients may experience postoperative complications that can cause significant morbidity and mortality.^19^ Despite advances in surgery, CPB and cardioplegic arrest techniques, the incidence of AF after cardiac surgery has increased significantly and remains relevant.^20^

POAF, which is a short-lived and self-limiting arrhythmia that usually begins two to four days after surgery, is an important complication with a frequency of 20–40% in cardiac surgical procedures and 10–20% in non-cardiac thoracic operations.^21^ Luo et al. reported that the incidence of POAF was 23.36% in patients aged 60 years and older who underwent isolated CABG with CPB.^22^ POAF most commonly occurs between the second and fourth postoperative days and is self-limiting in most patients.^9^ POAF is known to be a transient and benign complication of CABG, but recent studies have reported an association between POAF and increased early mortality and morbidity rates, including stroke, renal and respiratory failure, increased length of ICU and hospital stay, and hospital costs.^7^

Reducing POAF can result in significant savings; therefore, recently there has been increasing interest in identifying risk factors that could enable the implementation of preventative strategies.^9^ In our study, we found that POAF developed in 23.2% of patients after isolated CABG surgery, which is in accordance with the literature.

The exact pathophysiology of POAF after cardiac surgery has not been fully elucidated.^3^ Identified potential predisposing factors include age, presence of myocardial ischaemia, type of cardiac surgery, atrial distension and pre-existing cardiac conditions.^23^ Studies have shown that the mechanism of POAF includes intra-operative and postoperative phenomena such as inflammation, oxidative stress, sympathetic activation and cardiac ischaemia, and these combine to trigger AF.^21^

There are many studies investigating the relationship between clinical risk factors, demographic conditions, and pre-, peri- and post-operative biomarkers to predict the development of POAF in CABG patients.^24^ Recent biomarkers that support clinical outcomes and show solid evidence have broadened the medical field and helped clinicians in many specialities to predict the clinical course.^20^ Previous studies have shown biomarkers to be used as predictors of patient condition after cardiac surgery.^23^ In this study, we evaluated the effect of the pre-operative and early postoperative values of RDW, one of the recently used biomarkers, which has been shown to predict the incidence of POAF in patients without a previous history of AF who have undergone isolated CABG with CPB.

One potential cause of POAF is post-surgical inflammation, indicated by increased levels of inflammatory biomarkers such as CRP and Il-6.25 Although still a matter of debate, the role of these inflammatory biomarkers in the pathogenesis of POAF remains a subject of intense research.^23^ Anti-inflammatory agents used after CABG and/or valve surgery are an indication that inflammation plays a contributing role to POAF and reduce the incidence of AF.^21^

CRP and ESR are acute-phase reactants that increase in response to pro-inflammatory cytokines and other endogenous signals of innate immunity or tissue damage and are widely used as prognostic biomarkers in cardiovascular events.^26^ High pre-operative CRP levels were associated with POAF after CABG, but all studies emphasised that local stress and inflammation caused by surgery were more important than other causes of inflammation regarding POAF.^27^ Olesen et al. reported in their study that increased postoperative CRP levels after CABG were associated with POAF.^28^ Our study, which excluded patients with multiple risk factors for AF, showed high pre-operative and early postoperative CRP and ESR values to be independent risk factors for AF following isolated CABG.

PLR and NLR, which are widely used as inflammation biomarkers, are known to be associated with poor prognosis in cardiovascular disease.^12^ Gungor et al. reported in their study that age and high pre-operative PLR levels were independent predictors of AF after CABG.^29^ In their systematic review and meta-analysis, Liu et al. emphasised that high pre-operative NLR level was a promising prognostic biomarker in predicting POAF after cardiac surgery, but larger-scale validation studies are needed to confirm the integration of pre-operative NLR testing into routine clinical practice.^30^ However, PLR and NLR inflammation indices were not evaluated in this study.

RDW, which reflects the variability in the size of circulating red blood cells and is mostly used in the differential diagnosis of anaemia, is a new biomarker that has been associated with adverse cardiovascular events (myocardial infarction, atherosclerosis, heart failure, AF and left atrial thrombus) in many studies independently of other haematological indicators.^10^ Evidence from recent studies shows that RDW is a prognostic marker of AF in a variety of clinical settings.^15^

The first epidemiological study describing the relationship between RDW and AF was published in 2010 by Horne et al. In this prospective study, a total of 3 927 patients who underwent coronary angiography to define the frequency of cardiovascular disorders and complications (including AF) were evaluated after 30 days and one year, and an increased incidence of AF was observed with high RDW levels.^31^ Providencia et al. reported in their cross-sectional study that left atrial thrombus was significantly more common in patients with RDW ≥ 15.0% than in patients with a low RDW. This study included 247 patients who presented to the emergency department with symptomatic AF and underwent transoesophageal echocardiography to rule out left atrial appendage thrombus.^32^

In their retrospective study including 132 patients who underwent elective CABG surgery, Ertaş et al. reported that the risk of new-onset AF increased approximately 1.5 times in patients with RDW > 13.45%.33 Korantzopoulos et al. reported in their prospective study on 109 patients undergoing elective cardiac surgery that patients with RDW > 13.35% had a 46% higher risk of developing POAF during the hospital stay.^34^ Similarly, Pilling et al. reported that high RDW levels were associated with AF, and high RDW levels predicted new-onset AF in healthy volunteers.^35^ Jurin et al. reported in a recent study that RDW increased with enlargement of the left atrium and was independently associated with AF progression.^36^

In our study, the RDW levels in the pre-operative and early postoperative periods were significantly higher in the AF group compared to the non-AF group, and were consistent with previous studies. We also found that increased pre-operative and early postoperative RDW levels were an independent predictor for the development of early POAF in patients who underwent isolated CABG with CPB. In addition, we found that RDW levels were higher in patients with AF who had adverse events (such as stroke, heart failure, thromboembolism, mortality), which makes our study different from other studies. This result suggests that inflammation may be more active in patients in the AF group, reflecting increased RDW levels.

Previous studies have shown the association between coronary artery disease and some haematological parameters, including high RDW level.^37^ Osadnik et al. reported that high RDW values were an independent predictor of mortality in patients with stable coronary artery disease.^38^ Another study has shown that high RDW value was a strong predictor of mortality and major adverse cardiac events in patients presenting with acute coronary syndrome (ACS).^39^ Rosas-Cabral et al. reported in their study that increased volume of erythrocyte distribution was associated with short-term cardiovascular mortality in patients with ACS.^40^ In another meta-analysis study, Abrahan et al. reported that RDW was associated with mortality and risk of major cardiovascular events in patients diagnosed with ACS.^39^

Our study showed that AF occurring after cardiac surgery was closely associated with high pre-operative and early postoperative RDW levels. This biomarker, like others, has shown good performance in predicting new-onset AF following cardiac surgery, which may offer clinicians the possibility of early intervention and treatment to reduce the incidence of complications related to AF.

## Limitations of the study

A few limitations to our study should be mentioned. The primary limitation is that it included a limited, retrospective study population prone to prejudice, and it was a single-centre study, unlike multicentre and cross-sectional studies. Second, there are no follow-up results. Third, data from all patients were collected from this single centre, which may lead to biased results. Fourth, we could not observe time-dependent changes in RDW values and postoperative AF due to the retrospective study design. Accordingly, we could not evaluate the causal relationship between RDW values and the development of postoperative AF. Fifth, we did not evaluate RDW and other inflammatory biomarkers other than CRP and ESR in the patients included in the study. Sixth, the inflammatory process is dynamic and has persistence, but RDW levels were not dynamically measured multiple times in our study. Therefore, the dynamic trend and value of the indicator were not reflected in this study.

## Conclusion

We believe that RDW, which can be easily obtained from a simple complete blood count and is an inexpensive inflammatory marker, may be helpful in predicting the development of early POAF in patients who underwent isolated CABG with CPB. Although we were not able to establish a causal relationship in this study, some clinical implications may be obtained if the results of this study are confirmed by large-scale prospective studies.
